# Midkine-A functions upstream of Id2a to regulate cell cycle kinetics in the developing vertebrate retina

**DOI:** 10.1186/1749-8104-7-33

**Published:** 2012-10-30

**Authors:** Jing Luo, Rosa A Uribe, Sarah Hayton, Anda-Alexandra Calinescu, Jeffrey M Gross, Peter F Hitchcock

**Affiliations:** 1 Department of Ophthalmology and Visual Sciences, University of Michigan, W. K. Kellogg Eye Center, 1000 Wall Street, Ann Arbor, MI 48105-0714, USA; 2 Section of Molecular Cell and Developmental Biology, University of Texas at Austin, Austin, TX, USA

**Keywords:** Proliferation, Growth factors, Signaling pathways

## Abstract

**Background:**

Midkine is a small heparin binding growth factor expressed in numerous tissues during development. The unique midkine gene in mammals has two paralogs in zebrafish: *midkine-a* (*mdka*) and *midkine-b* (*mdkb*). In the zebrafish retina, during both larval development and adult photoreceptor regeneration, *mdka* is expressed in retinal stem and progenitor cells and functions as a molecular component of the retina’s stem cell niche. In this study, loss-of-function and conditional overexpression were used to investigate the function of Mdka in the retina of the embryonic zebrafish.

**Results:**

The results show that during early retinal development Mdka functions to regulate cell cycle kinetics. Following targeted knockdown of Mdka synthesis, retinal progenitors cycle more slowly, and this results in microphthalmia, a diminished rate of cell cycle exit and a temporal delay of cell cycle exit and neuronal differentiation. In contrast, Mdka overexpression results in acceleration of the cell cycle and retinal overgrowth. Mdka gain-of-function, however, does not temporally advance cell cycle exit. Experiments to identify a potential Mdka signaling pathway show that Mdka functions upstream of the HLH regulatory protein, Id2a. Gene expression analysis shows Mdka regulates *id2a* expression, and co-injection of Mdka morpholinos and *id2a* mRNA rescues the Mdka loss-of-function phenotype.

**Conclusions:**

These data show that in zebrafish, Mdka resides in a shared Id2a pathway to regulate cell cycle kinetics in retinal progenitors. This is the first study to demonstrate the function of Midkine during retinal development and adds Midkine to the list of growth factors that transcriptionally regulate Id proteins.

## Background

Neurogenesis relies on the coordinated interplay of intrinsic and extrinsic mechanisms that determine the spatial and temporal patterns of proliferation, initiate cell cycle exit, fix cell identities, and govern differentiation. The vertebrate retina is a long-standing model for investigating the mechanisms that govern developmental neurogenesis [1]. The retina has a limited number of cell types arrayed in evolutionarily highly conserved spatial patterns and functional circuits, and experimental alterations of retinal development are easy to detect by simple microscopic inspection. Cell-extrinsic signals govern retinal development by modulating intrinsic signals that can drive self-renewal, cell cycle exit, and differentiation. For example, growth factors at the midline divide the early eye field into two retinal anlagen that express unique combinations of transcription factors [2], and signaling centers both outside and inside the eye pattern the optic vesicle and initiate cell cycle exit and neuronal differentiation [1].

Midkine is a secreted heparin binding growth factor with a molecular weight of 13 kDa that is a member of the midkine/pleiotrophin family of growth factors [3]. Midkine is highly conserved among vertebrates and plays an important role in both development and disease. In mammals, midkine is highly expressed during embryogenesis, and down-regulated at birth. In the mammalian central nervous system (CNS), the expression of *midkine* is temporally and spatially regulated in a manner that suggests it functions to govern aspects of neurogenesis [3-7], although this has not been experimentally tested.

The unique *midkine* gene in mammals has two paralogs in zebrafish: *midkine-a* (*mdka*) and *midkine-b* (*mdkb*). In zebrafish, the two sub-functionalized genes have non-overlapping patterns of expression and independent biological functions [8-11]. In the embryonic and larval retina of zebrafish, *mdka* is expressed in retinal progenitors, but immediately down-regulated in these cells as they exit the cell cycle. During photoreceptor regeneration in the adult retina, both *mdka* and *mdkb* are up-regulated in Müller glia as these cells re-enter the cell cycle and adopt the features of retinal stem cells [10].

Here we establish that Mdka controls the cell cycle kinetics of retinal progenitors in the embryonic retina of zebrafish and functions upstream of the intrinsic HLH regulatory protein, Id2a. Following targeted knockdown of Mdka synthesis, retinal progenitors progress more slowly through the cell cycle, and this gives rise to microphthalmia, a diminished rate of cell cycle exit and delay of neuronal differentiation. In contrast, Mdka overexpression results in acceleration of the cell cycle and a transient retinal overgrowth, but Mdka gain-of-function does not temporally advance cell cycle exit. Gene expression analysis shows that Mdka regulates *id2a* expression, and co-injection experiments show that *id2a* mRNA rescues morpholino-induced Mdka loss-of-function. These data demonstrate that in the developing vertebrate retina the Mdka/Id2a pathway functions to regulate cell cycle kinetics and identifies Mdka as an extrinsic regulator of neurogenesis in the vertebrate central nervous system.

## Results

### Mdka knockdown transiently blocks neuronal differentiation

**Figure 1:**
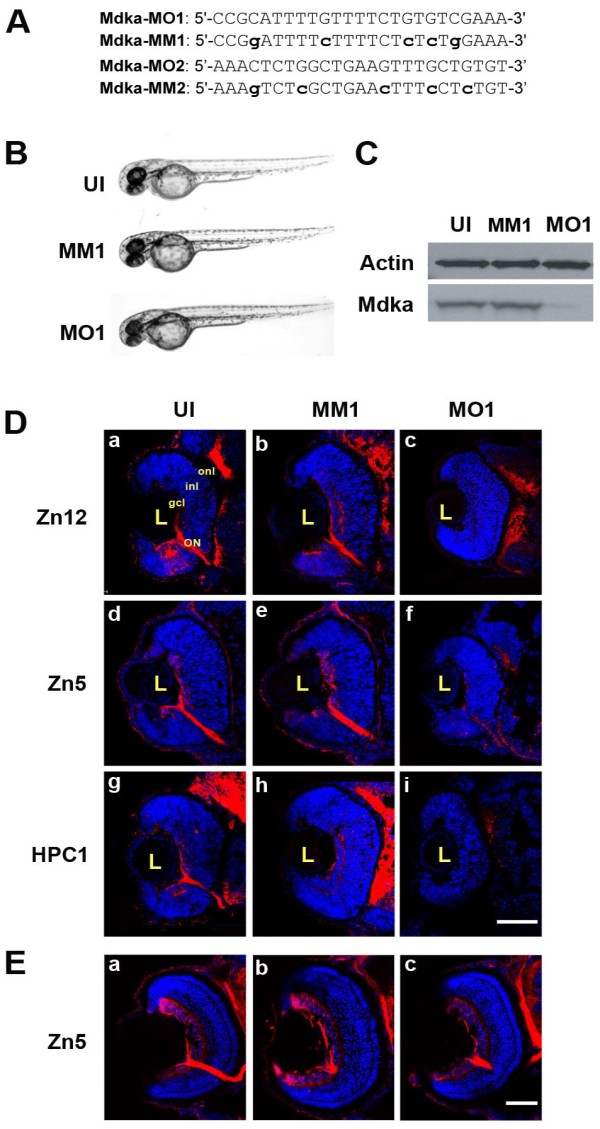
***Mdka *-targeted morpholinos inhibit Mdka synthesis and delay neuronal differentiation. (A)** Sequences of the two morpholino oligos (MO1, MO2) and the respective mismatch (MM) controls used in this study. **(B)** Representative embryos from the control and experimental groups at 48 hours post fertilization (hpf). **(C)** Western blot prepared from 48 hpf embryos. MM, embryos injected with 5-nucleotide mismatch control morpholinos; MO, embryos injected with MO1 *Mdka*- targeted morpholinos; UI, uninjected control. Note the absence of protein in embryos injected with the *mdka*-targeted morpholinos (similar results were observed using MO2.) The upper bands are stained with antibodies against actin and serve as loading controls. **(D)** (a, d, g) Sections taken through the retinas of uninjected embryos (UI) at 48 hpf and stained with antibody markers for ganglion cells (zn12; zn5) and amacrine cells (HPC-1), respectively. (b, e, h) Sections of retinas of embryos injected with 5-nucleotide mismatch morpholinos (MM1) and immunostained as above. (c, f, i) Retinas of embryos injected with Mdka translation-blocking morpholinos (MO1) and immunostained as above. Note the absence of differentiation markers that label inner retinal neurons. **(E)** Representative sections from uninjected (a), control (b), and loss-of-function embryos (c) allowed to survive to 72 hpf. The absence of neuronal differentiation at 48 hpf is recovered by 72 hpf. gcl, ganglion cell layer; inl, inner nuclear layer; L, lens; ON, optic nerve; onl, outer nuclear layer; . Scale bar equals 50μm. MIP 3D; Maximum intensity projection, three dimensional.

To inhibit Mdka synthesis, two non-overlapping *mdka* morpholinos (MO1, MO2; Figure 1A) were used. Control morpholinos contained five mismatched nucleotides (MM1, MM2; Figure 1A). At 48 hours post fertilization (hpf), embryos injected with the *mdka*-targeted morpholinos are indistinguishable from controls with respect to body size and shape, though experimental embryos display a mild, but statistically significant decrease in eye size (Figure 1B; uninjected (UI) *vs.* Mdka morpholinos, *P*=0.0002; MM morpholinos *vs.* Mdka morpholinos, *P*=0.001). Both MO1 (4 ng/embryo) and MO2 (5 ng/embryo) produced similar retinal phenotypes, and the specific suppression of Mdka protein synthesis was confirmed for MO1 (Figure 1C). All Mdka knockdown experiments presented here were performed using MO1.

Sections taken through the retinas revealed that at 48 hpf knockdown of Mdka results in small retinas, the absence of lamination and few overtly differentiated cells, which was confirmed using the antibody markers, zn5, zn12, and HPC1, which label inner retinal neurons (Figure 1D). Interestingly, however, the absence of neuronal differentiation (and lamination) was transient. Through 56 hpf, there was little neuronal differentiation following Mdka loss-of-function, whereas, by 72 hpf, even though western blot analysis showed that Mdka synthesis remains inhibited (data not shown), experimental retinas were largely normal with respect to the lamination and neuronal differentiation (Figure 1E).

### Mdka loss-of-function does not delay the onset of a neurogenic program or increase cell death

**Figure 2:**
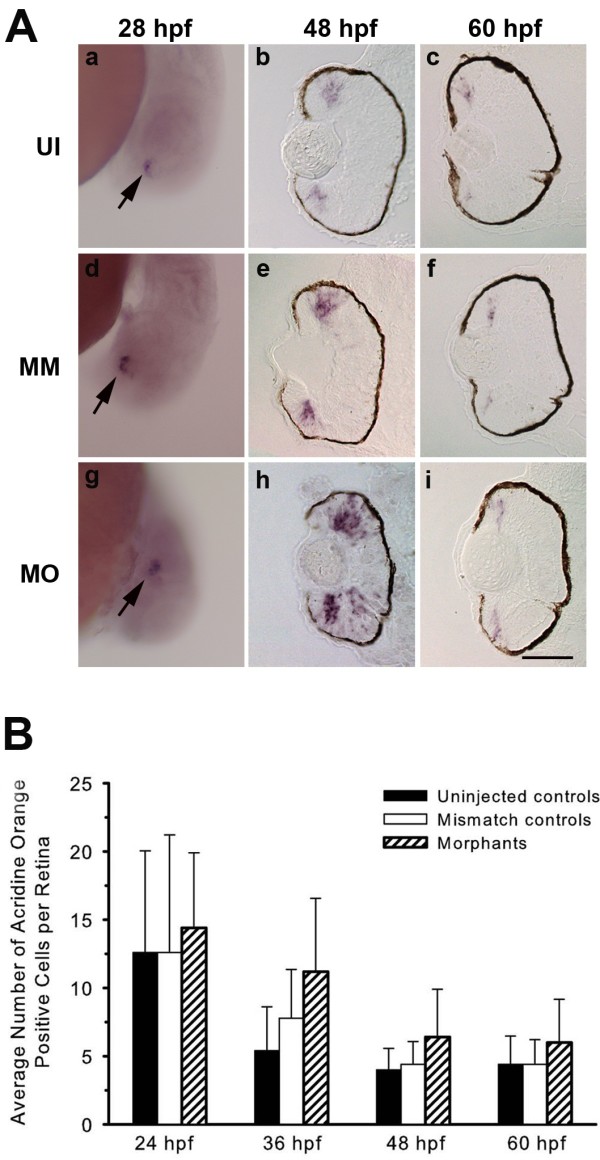
**Loss of Mdka function does not affect neurogenic competence or increase cell death. (A) ***In situ* hybridizations showing *atoh7* expression in the retinas of uninjected (a-c), control (d-f) and Mdka loss-of-function embryos (g-i). Arrows in panels a, d and g identify the initial cluster of *atoh7*-expressing cells in ventral retina. MM, embryos injected with 5-nucleotide mismatch morpholinos; MO, embryos injected with *mdka*-targeted morpholinos; UI, uninjected. Scale bar equals 50μm. **(B)** Average number of acridine orange-stained cells in the retinas of control and experimental embryos showing that loss of Mdka does not increase cell death. *P* >0.05 for all pairwise comparisons. *n*=5 to 7 embryos/time point. Scale bar equals 50 μm.

The delayed neuronal differentiation resulting from Mdka loss-of-function could result from four mechanisms, alone or in combination: (1) delay in the onset of the developmental neurogenic program; (2) apoptosis of newly post mitotic cells; (3) normal cell cycle exit but failure to differentiate; or (4) lengthening of the cell cycle, resulting in a temporal delay in cell cycle exit and, consequently, an absence of neuronal differentiation. In the vertebrate retina, the proneural gene *atoh7* (previously designated *ath5*) is an intrinsic timer required to initiate neurogenesis. The onset of expression of *atoh7* was used to test whether or not the loss of Mdka alters the initiation or maintenance of genetic programs required for neuronal differentiation. In zebrafish, expression of *atoh7* begins at approximately 25 hpf, within ventral-temporal retina and then expands through the retina as a wave spreading in a dorso-nasal direction, marking cells that are competent to exit the cell cycle and differentiate [12,13]. *In situ* hybridization analysis showed that the timing of the onset of *atoh7* expression is not altered in experimental embryos (Figure 2A, a-i), indicating that the absence of neuronal differentiation following Mdka knockdown does not result from simply delaying the onset of genetic programs that endow neurogenic competence. Further, the initial broad expression domain of *atoh7*[13] persists following Mdka knockdown (Figure 2A, h) whereas in control embryos *atoh7* mRNA becomes restricted to progenitors at the retinal margin (Figure 2A b,e). The persistence of the broad *atoh7* expression domain in the experimental retinas reflects the absence of cellular differentiation following Mdka knockdown and the neurogenic competence these cells retain (Figure 2A, h; see below).

Cell death in the developing zebrafish retina is normally very low [14]. The DNA-binding vital dye, acridine orange, was used to label pyknotic nuclei of dying cells in control and experimental embryos between 24 hpf and 60 hpf. Counts of acridine orange-labeled nuclei showed no significant differences in the number of dying cells in the retinas of control and experimental animals (Figure 2B). Terminal deoxynucleotidyl transferase dUTP nick end labeling (TUNEL) was also used to mark cells undergoing apoptosis, and no significant differences were observed in the number of TUNEL-positive cells in control and experimental groups (data not shown).

To test whether or not following knockdown of Mdka cells exit the cell cycle on time but merely fail to differentiate, embryos were given multiple injections of EdU between 34 hpf and 48 hpf and sacrificed at 48 hpf. DAPI-positive/EdU-negative (post-mitotic) cells were then counted in representative sections from experimental and control embryos (*n*=10 embryos/group) and averaged to determine the relative proportion of cells that had exited the cell cycle at 34 hpf prior to the availability of the EdU (see [15]). This analysis showed that relative to controls, following knockdown of Mdka, significantly fewer cells exit the cell cycle at 34 hpf (Table 1). This result indicates that loss of Mdka function does not delay neuronal differentiation, but, rather, alters the timing of cell cycle exit.

**Table 1:** Gain of Mdka function does not advance cell cycle exit

	**Average number of cells/section at 34 hpf**	**EdU-negative cells at 48 hpf (*n*)**	**Proportion of EdU-negative cells at 48 hpf**
Untreated	156 ± 22	53 ± 10	0.34 ± 0.09
Mdka l-o-f	138 ± 19	2 ± 2	0.02 ± 0.02^a^
WT	147 ± 23	51 ± 38	0.34 ± 0.17
Mdka g-o-f	191 ± 32	71 ± 28	0.38 ± 0.08

For each group, half the embryos were sacrificed at 34 hpf for cell counts. The remaining embryos were injected with EdU every 6 h starting at 34 hpf and fixed at 48 hpf. EdU-negative cells counted at 48 hpf were those that had exited cell cycle by 34 hpf. The proportion of EdU-negative cells was not significantly different between the untreated, WT, and Mdka g-o-f groups. The proportion of post-mitotic cells in the Mdka l-o-f group was significantly smaller than in controls. g-o-f, gain-of-function; l-o-f, loss-of-function. ^a^*P* <0.05.

### Mdka loss-of-function alters cell cycle kinetics

**Figure 3:**
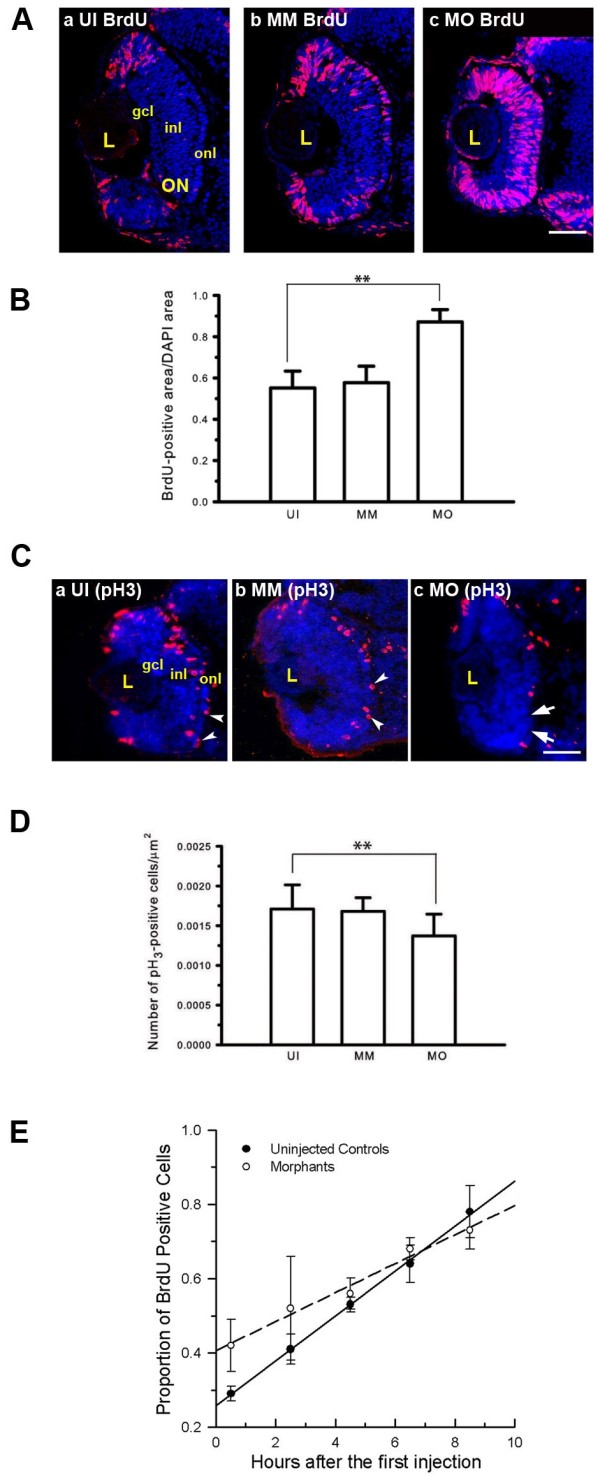
**Mdka loss-of-function alters cell cycle kinetics. (A)** BrdU labeled progenitors in uninjected (a) control (b) and Mdka loss-of-function embryos (c). **(B)** Histograms showing the average proportion of the retinal section occupied by BrdU-labeled cells. *P <0.001. *n* = 9 retinas/group. Scale bar equals 50μm*.***(C)** Retinas of embryos at 48 hpf stained with antibodies against phosphohistone H3 (pH3) in uninjected (a), control (b), and Mdka loss-of-function embryos (c). **(D)** The number of pH3-stained cells/unit area. ^**^P <0.01. *n*=12 embryos/group. MM, embryos injected with 5-pair mismatch control morpholinos; MO, embryos injected with *mdka*-targeted morpholinos (MO); UI, uninjected embryos. gcl, ganglion cell layer; inl, inner nuclear layer; L, lens; ON, optic nerve; onl, outer nuclear layer. Scale bar equals 50 μm. **(E)** Graph of the least-squares regression lines through data showing the proportion of BrdU-labeled progenitors as a function of time. An F-test was used to compare the control and experimental data. **P <0.05.

The absence of both neuronal differentiation and the normal timing of cell cycle exit imply that Mdka functions to control cell cycle kinetics. Therefore, a variety of approaches were used to evaluate cell proliferation and cell cycle kinetics among the retinal progenitors. This was investigated first by providing a brief systemic pulse of BrdU at 48 hpf and evaluating the spatial distribution and proportion of labeled cells immediately thereafter (Figure 3A). In control retinas the majority of proliferating cells are within the marginal germinal zone, and the proportion of the retina occupied by proliferating cells is low. In embryos injected with control morpholinos, there were extra BrdU-labeled cells in the central retina, and we interpret this to be a consequence of a simple developmental delay resulting from the injection procedures and/or presence of the morpholinos [16]. In contrast to control retinas, in the absence of Mdka function, at 48 hpf BrdU-labeled progenitors are present throughout the retina (Figure 3B), indicating that in central retina progenitors fail to exit the cell cycle at the appropriate developmental stage.

To further investigate the change of cell cycle kinetics following loss of Mdka function, the mitotic status of retinal progenitors was measured by counting progenitors labeled with antibodies that recognize the phosphorylated form of histone H3 (pH_3_), a marker specific for cells in the M-phase of the cell cycle (Figure 3C). In vertebrate retinas, cells undergo mitosis at the outermost (apical) surface of the retina, and this spatial pattern of pH_3_ labeling did not differ in control and experimental retinas. However, the proportion of pH_3_-positive cells in retinas lacking Mdka was significantly reduced relative to controls (Figure 3D).

To estimate the length of the cell cycle and the duration of the relative S-phase, we employed a cumulative BrdU labeling method [17]. This method provides a sustained systemic dose of BrdU and labels all cells passing through the S-phase of the cell cycle. BrdU was injected into the yolk at 2-h intervals beginning at 26 hpf, for 8-h. At 120-min intervals, three to four embryos from each group were removed, fixed, and the proportion of BrdU labeled cells was determined and plotted as a function of time. A linear regression was then fitted to these data. The slope and y-intercept of the best-fit lines were used to estimate the total length of the cell cycle and the relative duration of the S-phase, respectively. This analysis showed that the total length of the cell cycle was significantly longer for retinal progenitors following Mdka loss of function (21.50 h *vs.* 15.0 h; Figure 3E), and this could be accounted for largely by an increase in the duration of the S-phase (8.75 h *vs.* 4.10 h). Together, the pulse and cumulative BrdU labeling assays suggest that following knockdown of Mdka, retinal progenitors cycle more slowly, fail to exit the cell cycle at the appropriate developmental stage, and this results in the transient delay in neuronal differentiation.

### Mdka overexpression produces retinal overgrowth

**Figure 4:**
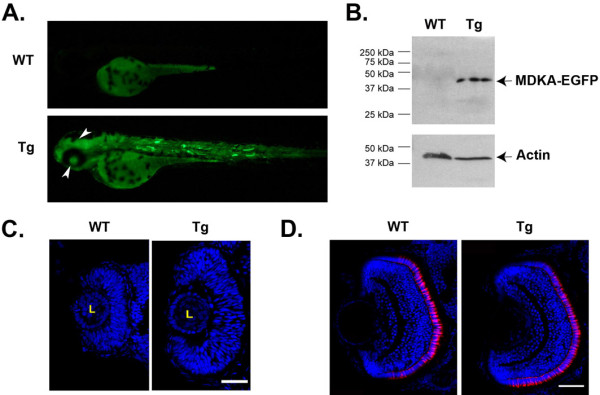
**Conditional over-expression of Mdka. (A)** Induced fluorescence in a Tg(pHSP70/4:*mdka:*egfp) larva at 72 hpf after heat shock treatment at 24 hpf. **(B)** Western blot stained with antibodies against EGFP showing induction of the fusion protein at 72 hpf after heat shock at 24 hpf. Tg, heat shock-treated, transgenic siblings; WT, heat shock-treated, wild-type embryos. **(C)** Mdka gain of function results in marked retinal overgrowth at 30 hpf, which is largely resolved by 72 hpf **(D)**. Tg, heat shock-treated embryos from the Tg(pHSP70/4:mdka:egfp) line; WT, heat shock-treated wild-type siblings. Scale bar equals 50 μm.

Transgenic fish were generated to conditionally induce Mdka overexpression *in vivo*. Three lines (kec1001L3, kec1004L4, kec1004L6) were identified that showed germ line transmission of the inducible *mdka/egfp* construct (pHSP70/4:*mdka:egfp*). When subjected to heat shock at 24 hpf, the Mdka-GFP fusion protein was visible in transgenic animals within 2 h, and wild-type, hemizygous, and homozygous offspring were easily distinguished based on GFP fluorescence intensity. Fluorescence imaging over time and western blot analysis using the anti-GFP antibody (the Mdka antibody does not recognize the fusion protein) demonstrated that the Mdka fusion protein is stable in transgenic embryos for at least 48 h (Figure 4A, B). Consistent with our model that Mdka governs cell cycle kinetics, Mdka gain-of-function in early embryos resulted in a marked retinal overgrowth (Figure 4C). Interestingly, similar to the results from Mdka loss-of-function, the retinal overgrowth observed in early embryos was also transient and largely resolved by 72 hpf (Figure 4D).

### Mdka levels alter the length of the G2 phase of the cell cycle

**Figure 5:**
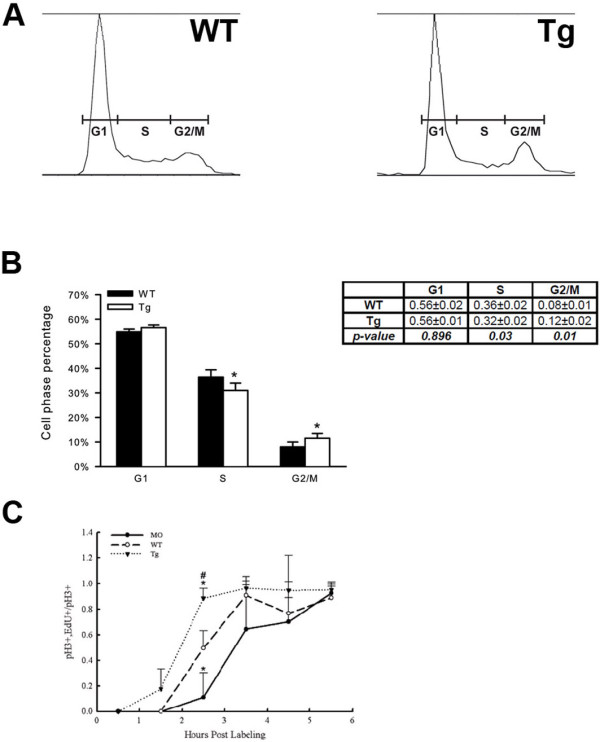
**Mdka gain-of-function accelerates the cell cycle. (A)** Representative raw data from FACS sorting of neural progenitors at 30 hpf. **(B)** Histograms showing the average percentage of cells in the G1-, S-, and G2-/M- phases of the cell cycle. *P <0.01. *n*=4 independent assays from a minimum of 5,000 cells/assay. **(C)** Graph showing the percent labeled mitoses for embryos between 28 hpf and 34 hpf. ^#^*P <0.01. *n*=4 embryos/condition/time point. MO, embryos injected with *mdka*-targeted morpholinos; Tg, homozygous transgenic siblings treated with heat shock at 24 hpf; WT, untreated wild-type embryos.

To directly evaluate cycling progenitors following Mdka gain-of-function, FACS DNA-content analysis was used to quantify the proportion of cells that occupy each phase of the cell cycle. This was performed at 30 hpf (6 h after heat shock), when the vast majority of retinal progenitors are still proliferating and the retina lacks post-mitotic cells [14]. Although cells from the forebrain and non-neuronal cells surrounding the head and eyes are included in this assay, the FACS analysis showed that Mdka gain-of-function results in a statistically significant decrease in the proportion of cells in the S-phase, which was accompanied by a significant increase in the proportion of cells in G2/M. The proportion of cells in the G1-phase was unchanged (Table 1; Figure 5A, B).

Analyzing DNA content provides only the relative proportion of cells in each phase of the cell cycle. Therefore, the percent labeled mitoses paradigm (PLM; [18]) was used to complement the FACS analysis and directly measure the length of the G2-phase of the cell cycle among retinal progenitors. These experiments were performed using embryos subjected to heat shock at 24 h, sorted by genotype and evaluated between 28 hpf and 34 hpf. Due to the nature of this experiment, we were able also to include embryos injected with *mdka* morpholinos in the PLM analysis. As anticipated, the percentage of pH_3_/EdU double-labeled nuclei increased sigmoidally with time, demonstrating that the experimental treatments did not affect the ability of dividing cells to progress from the S- to M-phase of the cell cycle. Further, qualitative observations showed that as progenitors progress through G2 they physically moved from the inner retina to the apical surface, where they become coincident with pH3-positive cells [19]. Although variable, the data also showed a significant difference between the three groups at the 2.5-h time point, where nearly 100% of the progenitors with elevated Mdka had completed G2, compared to only approximately 10% of the progenitors in retinas lacking Mdka (Figure 5C). The values for wild-type cells were intermediate between these two. These data show directly that Mdka levels regulate progression from S- to M-phase of the cell cycle. The acceleration of the cell cycle following gain of Mdka function both accounts for the retinal overgrowth resulting from Mdka gain-of-function (Figure 4C) and is complementary to the consequences of Mdka loss-of-function (see above).

In an effort to explore the molecular mechanisms through which Mdka governs cell cycle kinetics, we used *in situ* hybridization to evaluate the relative expression levels of core cell cycle components, the cyclin genes, *B1*, *D1*, *E1*, and the CDK gene, *p27*[20,21]. There were no apparent differences in the relative expression levels or tissue distribution of these genes among untreated, loss-of-function, or gain-of-function embryos (data not shown), suggesting that Mdka does not modulate the duration of the cell cycle by directly regulating the expression of cell cycle kinases or kinase inhibitors.

### Gain of Mdka function does not advance cell cycle exit

Experiments were undertaken next to determine if elevating Mdka levels also accelerates cell cycle exit. Clutches from hemizygous transgenic crosses were treated by heat shock at 24 hpf, sorted into wild-type and homozygous transgenic groups, given multiple injections of EdU between 34 hpf and 48 hpf, and sacrificed at 48 hpf. DAPI-positive/EdU-negative (post-mitotic) cells were counted and averaged to determine the relative proportion of cells that had exited the cell cycle at 34 hpf prior to the availability the EdU (see [15]; see above). The average number of cells/retinal section was greater following Mdka gain-of-function, reflecting the early retinal overgrowth. However, elevating Mdka levels did not significantly increase the proportion of post mitotic (EdU-negative) cells over wild-type controls at 34 hpf (34% ± 17% *vs.* 38% ± 9%; Table 1), demonstrating that the Mdka-induced acceleration of the cell cycle does not also temporally advance cell cycle exit.

### Mdka functions upstream of the HLH regulatory protein Id2a

**Figure 6:**
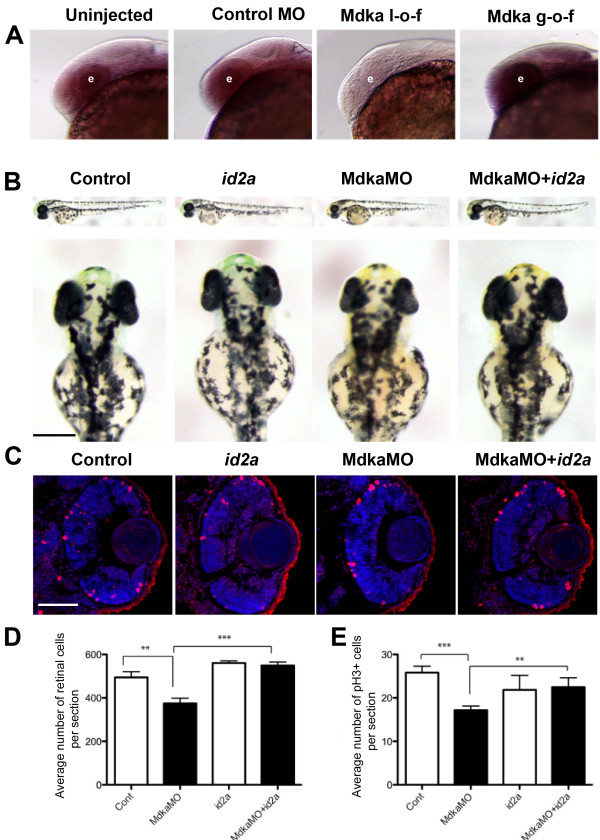
**Mdka functions upstream of Id2a. (A)** Wholemount *in situ* hybridizations showing *id2a* expression at 28 hpf. Embryos were uninjected (Uninjected), injected with mismatch Mdka morpholinos (Control MO), Mdka translation-blocking morpholinos (Mdka l-o-f), or transgenic embryos treated with heat shock at 24 hpf (Mdka g-o-f). The letter ‘e’ identifies the eye. Note that *id2a* expression is modulated by Mdka levels. **(B)** Representative control and experimental embryos at 48 hpf. Embryos were uninjected (Control), injected with *id2a* mRNA (*id2a*), Mdka translation-blocking morpholinos (MdkaMO), or co-injected with Mdka translation-blocking morpholinos and *id2a* mRNA. **(C)** Representative retinal sections from embryos at 48 hpf. The conventions are as above. Sections are stained with DAPI to label nuclei and antibodies against phosphohistone H3. **(D)** Graph showing the number of retinal cells in each group. **(E)** Graph showing the number of phosphohistone H3-positive cells in each group. Note that the number of DAPI and phosphohistone H3-positive cells are reduced following Mdka l-o-f and cell numbers are restored following co-injections of Mdka translation-blocking morpholinos and *id2a* mRNA. **P <0.05. Scale bars in panel B and C are 200 μm and 50 μm, respectively.

**Figure 7:**
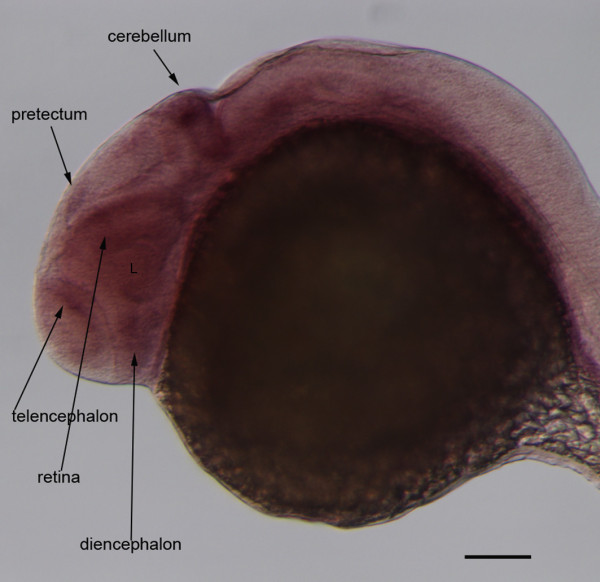
**Expression of *id2a *at 28hpf.***In situ* hybridization of wholemount embryos at 28 hpf recapitulates the tissue-specific pattern of *id2a* expression (cf. Figure 3, Thisse *et al*., 2004). Scale bar equals 100 μm L, lens.

The observations from the Mdka gain- and loss-of-function experiments strongly resemble data reported recently following gain- and loss-of-function of the HLH protein, Id2a [22]. In the early larval retina of zebrafish, Id2a is expressed in retinal progenitors where it functions as a non-cell autonomous regulator of cell cycle kinetics. Knockdown of Id2a lengthens the cell cycle, delays neuronal differentiation, and produces microphthalmia, whereas Id2a over-expression accelerates the cell cycle, leading to retinal overgrowth. The phenotypes associated with the gain and loss of Id2a persist through 72 hpf [22]. Gene expression studies were performed with embryos at 28 hpf to determine if there is a genetic relationship between Mdka and Id2a. First, *mdka* expression was evaluated by *in situ* hybridization following both the loss and gain of Id2a function. Altering Id2a levels did not alter *mdka* hybridization signal (data not shown). Second, *id2a* expression was evaluated by *in situ* hybridization following Mdka gain- and loss-of-function. For this experiment, the color reactions were stopped simultaneously for each group at a point where differences in the signal intensity between loss- and gain-of-function embryos became distinct. This resulted in an apparent incomplete pattern of *id2a* expression in the control embryos (Figure 6A). However, in an independent experiment, it was confirmed that using the same riboprobe and developing the color reaction to a sufficient degree recapitulates the expression pattern specific for id2a (Figure 7; cf. Figure 3 in Thisse *et al*., 2004 [23]). The comparative *in situ *hybridization analysis showed that relative expression of *id2a* is markedly reduced or lost following Mdka knockdown, whereas, relative *id2a* expression is increased following Mdka overexpression (Figure 6A). Third, quantitative Reverse Transcriptase-PCR (qRT-PCR) was performed on mRNA isolated from heads of embryos at 28 hpf following Mdka gain- and loss-of-function. This analysis showed that Mdka knockdown resulted in a 1.75-fold decrease in the expression of *id2a* (MM morpholinos *vs.* Mdka morpholinos, *P*=0.03), whereas Mdka overexpression resulted in a 5.14-fold increase in the expression of *id2a* (WT-heat shock *vs.* Tg-heat shock, *P*=0.018). Collectively, these data were interpreted to show that in the zebrafish retina Mdka functions upstream of Id2a and that Mdka levels govern the expression of *id2a*. To further test this model, Mdka morpholinos and *id2a* mRNA were co-injected into embryos and proliferation and neurogenesis in the retinas were analyzed. These experiments showed Id2a overexpression is sufficient to restore normal retinal development following Mdka knockdown. *id2a* rescues the microphthalmia (Figure 6B), restores the number of retinal cells to control levels (Figure 6C-E), and restores the normal timing of cellular differentiation (data not shown). These data show that in the zebrafish retina Mdka and Id2a function within a common signaling pathway that regulates retinal development.

## Discussion

### Mdka regulates cell cycle kinetics

Precisely regulated cell proliferation is a fundamental determinant of organ growth. Both intrinsic and extrinsic signaling molecules govern the kinetics of proliferation and the timing of re-entry into or exit from the cell cycle [24]. Relatively few studies have directly addressed the role of Midkine in governing cell cycle kinetics. During fetal CNS development, Midkine is necessary to maintain the M-phase of neural progenitor cells [25], and transfection of drug-sensitive cells with the midkine gene releases the drug-induced proliferation arrest and allows cells to progress through the S-phase [26]. More recently, midkine was shown to promote the growth of murine embryonic stem cells by preventing apoptosis and inducing the G1-S phase transition [7], and an *in vitro* assay showed that neurospheres from Mdka deficient mice are significantly smaller than those from wild types [27]. The earlier study from our lab showing that *mdka* is expressed in retinal progenitors [10] led to the hypothesis that in the zebrafish retina Mdka functions to regulate aspects of neurogenesis. We used loss- and gain-of-function approaches and assays of proliferation and cellular differentiation to test this hypothesis. The resulting data showed that Mdka functions in retinal progenitors to regulate kinetics of the cell cycle. Loss of Mdka significantly increases the duration of the cell cycle, which is manifested by a decreased number of mitotic cells and microphthalmia. Complementing these results, Mdka overexpression significantly accelerates the cell cycle, leading to a retinal overgrowth. Further, while both morpholino-induced knockdown and induced overexpression of Mdka synthesis persists until at least 72 hpf, the knockdown phenotype observed at 48 hpf was recovered by 60 hpf and the induced retinal overgrowth is largely resolved by 72 hpf. These data suggest that Mdka function in the embryonic retina predominates only during a relatively brief temporal window and during this time functions narrowly to govern cell cycle kinetics. Numerous extrinsic molecules regulate proliferation of vertebrate retinal progenitors, including Notch, FGF, BMP, Wnt, and HH (reviewed in [1]). Mdka can now be added to this list. Finally, *midkine* was identified as a component in the core transcriptional repertoire of mitotic progenitors in the mammalian retina and brain [4]. In light of these data, we propose that in the vertebrate central nervous system Midkine is a component of the complex environment of extrinsic regulatory molecules (for example [24]) that functions, perhaps in an autocrine manner, to regulate developmental neurogenesis.

### Mdka does not directly govern cell fate determination or neuronal differentiation

Lineage studies show that retinal progenitor cells give rise to neuronal and glial cell types in a characteristic order of birth [15,28,29]. Whereas Mdka knockdown slows the onset of neuronal differentiation, overexpression of Mdka does not advance cell cycle exit. These data suggest that the delay in neuronal differentiation following Mdka loss-of-function is a consequence of slowing the cell cycle, and Mdka does not normally function to promote neurogenesis. Similarly, Mdka gain-of-function accelerates the cell cycle, but does not also advance cell cycle exit and neurogenesis. The non-correlated effects between cell cycle regulation and cell-fate determination were also observed in the retina of disarrayed mutant zebrafish [30]. These observations, along with the data from our experiments, suggest that Mdka functions, perhaps narrowly, to regulate cell cycle kinetics without influencing cell fate specification or cellular differentiation. This stands in contrast to other intrinsic and extrinsic regulatory molecules, which have been shown both to coordinate cell cycle progression and cell fate determination [1].

### Mdka functions upstream of Id2a

The striking similarity in the data regarding Mdka function and those published recently describing the function of the protein, Id2a [22], led us to investigate the potential genetic relationship between Mdka and Id2a. Id (inhibitor of differentiation) proteins are intrinsic components of signaling pathways that function as positive regulators of cell cycle progression in neuronal progenitors [31] and key mediators of tumor progression in transformed cells [32,33]. Id proteins lack a basic, DNA-binding domain, and heterodimers formed between Ids and bHLH transcription factors cannot bind DNA or form active dimers (see [32]). Similar to Mdka, in zebrafish retinal progenitors, Id2a modulates S-phase progression and/or the S- to M-phase transition [22]. Loss of Id2a function lengthens the cell cycle, leading to microphthalmia and an absence of neuronal differentiation, whereas gain of Id2a function shortens the cell cycle, leading to retinal overgrowth. The similarities of the Mdka and Id2a functional studies suggested the hypothesis that in the retina Mdka and Id2a function in a shared development pathway.

Gene expression analysis and mRNA rescue experiments showed that in the developing retina Mdka functions upstream of Id2a within a common signaling pathway. Mdka is required for sustained *id2a* expression, and Mdka gain-of-function is sufficient to increase the transcription of *id2a*. Id2a has previously been shown to regulate retinoblast cell cycle kinetics, therefore, these data suggest that Mdka functions through Id2a to govern cell cycle control in the embryonic retina. This was confirmed by experiments showing that overexpression of *id2a* was sufficient to restore cell division and eye growth to normal levels following Mdka loss-of-function. In the developing retina of mammals, Bmp signaling is upstream of Id expression. In the retina, BMPs promote differentiation of retinal progenitors, and altering BMP4 upregulates the expression of multiple Id proteins [34]. The current study demonstrates that Mdka also modulates Id gene expression and suggests, therefore, that in the mammalian retina and brain Midkine may also be a component of signaling events mediated by Id proteins.

**Figure 8:**
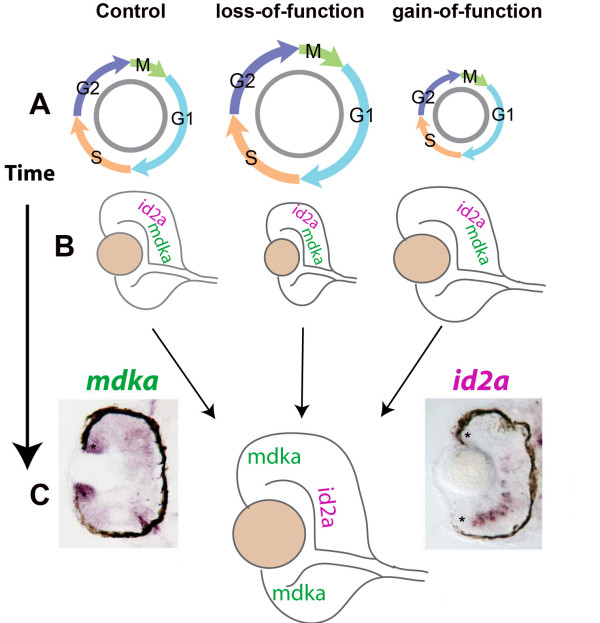
**Summary diagram.** The level of Mdka synthesis systematically alters the length of the cell cycle, as indicated by the diameter of the cell cycle cartoons **(A)**, and the size of the eye **(B)**. This corresponds also to the early expression of both *mdka* and *id2a* in retinal progenitors (**B**). By 48 hpf, *mdka* and *id2a* are differentially expressed in retinal progenitors and INL neurons, respectively, which is most evident for cells at the retinal margin (asterisks, **C**).

The knockdown and overexpression experiments show that Mdka function is transient and restricted to early retinal development. Retinas from embryos with altered Mdka levels recovered to resemble wild-type retinas by 72 hpf. This is in contrast to Id2a loss- and gain-of-function, which persists through 72 hpf. There are several possible explanations for the transient retinal phenotypes that result from manipulating Mdka levels. First, homeostatic mechanisms within the retina may compensate for perturbations of Mdka signaling in the early in retinal development. Second, the functional reduction and overexpression of Mdka may be transient. Morpholino dilution beyond 48 hpf may allow sufficient levels of Mdka synthesis to allow neurogenesis to resume, and the early pulse of Mdka overexpression following heat shock at 24 hpf may not be sufficient to maintain the cell cycle compression and retinal overgrowth through 72 hpf. Third, we speculate that the transient effects of altering Mdka levels reflect the divergent patterns of cellular expression for these two genes. In the embryonic retina, prior to the onset of neuronal differentiation (see [15]) both *mdka* and *id2a* are expressed in mitotic retinal progenitors ( [9]; [35]; [22]). However, by 48 hpf, *id2a* is down-regulated in mitotic cells and up-regulated in a population of post-mitotic cells in the inner nuclear layer (Figure 8). The segregation of the cellular expression indicates that the expression of *mdka* and *id2a* are differentially regulated and, thereby, may only transiently share a common regulatory pathway within progenitor cells during early retinal development. At around 48 hpf, a separate mechanism down-regulates the expression of *id2a* in retinal progenitors, which potentially disconnects Mdka-*id2a* signaling. This raises the possibility that Mdka/Id2a signaling functions in the early retina only. When these two signaling proteins are decoupled by differential patterns of expression, cellular differentiation may occur even in the absence of Mdka.

## Conclusions

The zebrafish genome encodes two Midkine paralogs, *mdka* and *mdkb*[8]*.* The potential role for Mdka in the retina was identified in a screen for genes in zebrafish retina that are up-regulated following photoreceptor death and during stem cell-based photoreceptor regeneration [10]. In the embryonic and larval CNS, the two *midkine* paralogs are differentially regulated, and each exhibits distinct temporal and spatial patterns of cellular expression [8,10]. Interestingly, in the retina, the expression of both midkine paralogs is induced in the intrinsic stem cells, Müller glia, as they re-enter the cell cycle in preparation for regenerative neurogenesis [10]. In the present study, we showed that in the embryonic retina of zebrafish Mdka functions to regulate cell cycle kinetics in retinal progenitors and this function is mediated through the transcriptional regulation of *id2a*. These data for the embryonic retina suggest that the Mdka/Id2a pathway may also function during the stem cell-based neuronal regeneration in the adult zebrafish retina.

## Methods

### Reagents

Nitrocellulose membranes, mouse anti-HPC-1, anti-goat peroxidase-conjugated secondary antibodies, propidium iodide (PI), and bromodeoxyuridine (BrdU) were purchased from Sigma (St. Louis, MO, USA). Anti-zpr1, anti-zpr3, anti-zn12, and anti-zn5 antibodies were purchased from the Zebrafish International Resource Center (ZIRC, Eugene, OR, USA). TOPRO3, Alexa Fluor 555 goat anti-rabbit IgG antibody, Alexa Fluor 555 goat anti-mouse IgG antibody, Alexa Fluor 488 goat anti-rabbit IgG antibody, the MultiSite Gateway Three-Fragment Vector Construction Kit, Click-it EdU Alexa Fluor 555 Imaging Kit, and trypsin were purchased from Invitrogen (Carlsbad, CA, USA). Rabbit anti-phosphohistone 3 (pH_3_) antibodies were purchased from Millpore (Billerica, MA, USA). Mouse anti-BrdU antibody was purchased from BD Biosciences (Franklin Lakes, NJ, USA).

### Experimental animals

AB strain zebrafish were purchased from Aquatic Tropicals Inc. (Bonita Springs, FL, USA) and housed at 28.5°C on a 14/10-h light/dark cycle. Embryos were collected after natural spawns, incubated at 28.5°C and staged by hpf [36].

### Morpholino oligo and mRNA injections

For loss-of-function experiments, two morpholino oligos (Gene Tools, LLC, Cowallis, OR, USA) were used that were targeted to non-overlapping sequences in proximity to the translation start site of the zebrafish *mdka* mRNA (NCBI Reference Sequence: 131070.2). Control morpholinos contained a 5-nucleotide mismatch. Morpholinos were diluted in 1× Danieau buffer [16] at 1 mg/mL, and embryos were injected with 2 ng to 5ng of morpholinos at the 1- to 8-cell stage.

For the mRNA rescue injections, 80 pg of *id2a* mRNA in a pCS2-id2a vector was co-injected with 5 ng of Mdka morpholino (see [22]).

### Western blot analysis

Mdka and GFP were detected in western blots using techniques described previously [9]. Proteins were extracted from pools of 15 to 20 embryos by lysing the embryos in buffer with protease inhibitors (Complete Mini, Roche, Germany). Proteins were separated in a 12% SDS-PAGE gel and transferred to a nitrocellulose membrane. The membrane was blocked in 5% non-fat dry milk in PBS for 2 h and incubated with rabbit anti-Mdka antibody (1:300; [9]) or rabbit anti-GFP antibody (1:500, Abcam, Cambridge, MA, USA). Blots were rinsed with PBS and incubated with goat horseradish peroxidase-conjugated secondary IgG (1:5,000) for 1 h. Bound antibodies were visualized using the enhanced chemiluminescence assay (ECL detection system, Amersham Biosciences, Arlington Heights, IL, USA). As loading controls, blots were stripped and incubated with anti-actin (1:1,000, Calbiochem, Germany).

### Immunohistochemistry

Embryos were fixed overnight in 4% paraformaldehyde dissolved in 100 mM phosphate buffer, cryoprotected by infiltration in 20% sucrose in phosphate buffer, and frozen in Optimal Cutting Temperature (OCT) media. Cryosections at 7 to 10 μm in thickness were mounted on glass slides and processed for immunohistochemistry using standard procedures. Briefly, after drying, sections were incubated with 20% normal sheep serum in phosphate buffered saline and 0.5% triton X-100 (PBST), followed by overnight incubation at 4°C with primary antibodies. After washing with PBST, sections were incubated in secondary antibodies for 1 h at room temperature, washed extensively in PBST. Sections were counterstained with 1:1,000 dilution of DAPI to label nuclei and sealed with mounting media and glass coverslips.

### Systemic labeling with BrdU or EdU

Proliferating cells were labeled with either BrdU or EdU by soaking embryos for 20 min in ice-cold 5 mM BrdU or 1.5 mM EdU dissolved in embryo rearing solution containing 15% DMSO. For BrdU staining, sections were incubated in 4 N HCl and immunolabeled using a mouse anti-BrdU antibody that was visualized with goat anti-mouse IgG antibodies conjugated to Alexa Fluor 555 [37]. EdU was visualized using the Click-iT™ EdU imaging kit with Alex Fluor 555 according to the manufacturer’s protocol.

### *In situ* hybridization

*In situ* hybridization on retinal sections was performed using digoxigenin (DIG)-labeled riboprobes, synthesized as previously described [37]. Briefly, sections were hybridized with probes overnight at 55°C. The next day, the sections were washed and incubated with an alkaline-phosphatase-conjugated anti-DIG antibody overnight at 4°C. After washing, riboprobes were visualized using 4-nitrobluetetrazolium/5-bromo-4-chloro-3-indolyl phosphate (NBT/BCIP; Roche, Indianapolis, IN, USA) solution as the enzymatic substrate (Roche).

For whole mount *in situ* hybridizations, embryos were fixed in 4% paraformaldehyde, dehydrated in methanol and stored at -20°C for a minimum of 12 h. Embryos were then returned to room temperature, rehydrated, fixed again in 4% paraformaldehyde, permeabilized with 0.1 M proteinase K, fixed a third time in 4% paraformaldehyde, treated with acetic anhydride, and washed in PBS with 0.1% Tween. Embryos were next washed in pre-hybridization buffer, which was removed and replaced with 500 μL of hybridization solution containing 200 ng of probe. Embryos were hybridized overnight at 55°C. The next day, embryos were washed and the digoxigenin was detected using antibodies conjugated to alkaline-phosphatase and a colorimetric reaction with NBT/BCIP as the enzymatic substrate. Embryos were transferred to single concavity slides for inspection and photomicroscopy.

### Acridine orange labeling

Apoptotic nuclei were visualized by labeling live embryos with the vital exclusion dye, acridine orange. At 24 hpf, embryos were transferred to 0.003% 1-phenyl-2-thiourea (PTU) to block melanin synthesis. At various ages, embryos were then dechorionated and placed in acridine orange solution (5 μg/mL in embryo medium; Molecular Probes, Eugene, OR, USA) for 15 min followed by extensive washes in embryo medium. Embryos were lightly anesthetized and viewed under a fluorescence dissecting scope, and acridine orange-positive cells were counted.

### Transgenic lines

The Gateway-based *Tol2*kit system was used to generate transgenic fish [38]. Mdka cDNA (pCS2p-mdk1; a gift from C. Winkler), was PCR-amplified with primers containing an attB1 site on the forward primer and a reverse attB2 site on the reverse primer. The sequence of these primers is as follows: Forward: 5^′^ GGGGACAAGTTTGTACAAAAAAGCAGGCTGTATGCGGGGCCTGTTTTCCACC 3^′^; Reverse: 5^′^ GGGGACCACTTTGTACAAGAAAGCTGGGTCGTTCCCTTTCCCCTTGCCTT 3^′^. The purified PCR products with added att sites were used immediately in BP reactions to generate middle entry clones. Multisite recombination reactions were performed to generate the pHSP70/4:*mdka:egfp* expression construct.

The expression construct and *in vitro* transcribed Tol2 transposase mRNA were co-injected into AB wild-type embryos at the 1- or 2-cell stage. Embryos positive for the gene construct were identified at 72 hpf by the HSP70/4-driven expression of EGFP in the lens ( [39]). Positive F0 fish were outcrossed to wild-type AB fish. F1 generation carriers were identified by GFP expression following heat shock and validated by PCR with genomic DNA, using primers for the sequence encoding the enhanced green fluorescent protein. Three lines, kec1001L3, kec1004L4, kec1004L6, were identified and used to propagate stable (Tg(pHSP70/4:*mdka:egfp*)) lines. These lines were characterized and found to yield approximately 50% GFP-positive progeny when mated to wild-type AB fish, suggesting a single Tol2 insertion. One transgenic line, Tg(pHSP70/4:*mdka:egfp*)^kec1001L3^, was used for all of the data presented here.

### Flow cytometry

Embryos were collected from pairwise, hemizygous x hemizygous matings and treated by heat shock (37°C for 60 min) at 24 hpf. Wild-type and homozygous transgenic embryos were identified at 26 hpf by the absence or presence and relative intensity of GFP. At 30 hpf, 50 heads (forebrains and eyes) from each group were isolated and washed twice in ice-cold PBS and dissociated into single-cell suspensions in 5 mL of ice-cold 0.25 mg/mL trypsin solution for 10 min at room temperature. Cells were fixed in ice-cold 70% ethanol for 30 min and stained with propidium iodide. DNA content for the isolated cells was analyzed on a FACScan II (Becton-Dickinson), and histograms of DNA content were acquired by CellQuest (BD Biosciences) and MODFIT LT (Verity Software House, Topsham, ME, USA).

### Cumulative BrdU labeling

To estimate the total length of the cell cycle and the length of the S-phase, a cumulative BrdU labeling approach was used [17,40]. BrdU was injected into the yolk at 2-h intervals beginning at 26 hpf, which provides a sustained systemic dose of BrdU and labels all cells passing through the S-phase of the cell cycle. At 30 min after each injection, three to four embryos from each group were removed, dechorionated, and fixed for BrdU immunohistochemistry. At each time point, DAPI- and BrdU-labeled nuclei were counted in retinal sections, and the proportion of cells labeled with BrdU was determined and plotted as a function of time. A least-squares regression line was then fit to the data.

### Percent labeled mitoses

The length of the G2-phase of the cell cycle was measured using the percentage labeled mitoses paradigm [18]. At 24 hpf, embryos were placed in embryo solution containing PTU (see above). At 28 hpf, embryos were incubated in the EdU/DMSO solution for 20 min then removed and returned to embryo solution. Ten minutes following the EdU exposure, and at 1-h intervals, subsequently, four embryos from each group were removed, dechorionated, and fixed. Whole embryos were stained with antibodies against phosphohistone H3 (pH_3_), followed by EdU labeling chemistry. Nuclei were counterstained with 1:1,000 dilution of TOPRO3. Retinas from whole embryos were imaged using a Leica upright confocal microscope (Leica DM6000 CFS) with 25× water immersion objective. Approximately 40 to 80 optical sections, 1 μm in thickness, were acquired for each eye. By surveying each optical slice every pH_3_-positive cell was counted and scored for the presence/absence of EdU.

### qRT-PCR

mRNA was isolated from the forebrains and eyes of embryos at 28 hpf following either morpholino injections (mismatch morpholinos *vs.* Mdka-targeted morpholinos) or heat shock treatment (wild type *vs.* Tg(pHSP70/4:*mdka:egfp*)^kec1001L3^ from a single clutch). Real-time PCR was performed using Power SYBER Green PCR Master Mix (Applied Biosystems) on the Applied Biosystems 7900HT Real Time PCR machine. Real-time PCR data were analyzed using the Comparative C_t_ method [41]. Fold changes in expression are normalized to tubulin levels. The following primer pairs were used: *id2a* forward 5^′^ GCATCCTCTCACTACAGACACC 3^′^, *id2a* reverse 5^′^ CCTGATTAACGGTAAAGTGTCCT 3^′^; *tubulin* forward 5^′^ TGGAGCCCACTGTCATTGATG 3^′^, *tubulin* reverse 5^′^ CAGACAGTTTGCGAACCCTATCT 3^′^.

### Statistical analysis

All quantitative data are represented as means and standard deviations. Statistical significance between groups was determined either by a one-way ANOVA or a Student’s t-test using GraphPad Prism software (GraphPad Software, La Jolla, CA, USA). *P* values <0.05 were considered statistically significant.

### Imaging

Images of sectioned retinas were captured using a Leica TCS SP5 confocal microscope (Vernon Hills, IL, USA).

## Abbreviations

*atoh7*: mRNA encoding atonal homologue 7; bHLH: Basic helix-loop-helix; BMP: Bone morphogenetic protein; BrdU: 5-bromo-2'-deoxyuridine; CNS: Central nervous system; DAPI: 4',6-diamidino-2-phenylindole; EdU: 5-ethynyl-2'-deoxyuridine; *egfp*: mRNA encoding enhanced green fluorescent protein; FACS: Fluorescence-activated cell sorting; FGF: Fibroblast growth factor; GFP: Green fluorescent protein; g-o-f: Gain of function; HLH: Helix-loop-helix proteins; hpf: Hours post fertilization; Id2a: Inhibitor of differentiation protein; HH: Hedgehog protein; *id2a*: *mRNA encoding* Id2a; l-o-f: Loss of function; Mdka: Midkine-a protein; *Mdka*: mRNA encoding Midkine-a; *mdkb*: mRNA encoding Midkine-b; MO: Targeted morpholino oligonucleotides; MM: Mismatch morpholino oligonucleotides; mM: milimolar; mRNA: messenger RNA; PBS: Phosphate buffered saline; PCR: Polymerase chain reaction; pg: Picograms; pH_3_: Phosphorylated histone H3; PLM: Percent labeled mitoses; PTU: 1-phenyl-2-thiourea; qRT-PCR: Quantitative reverse transcriptase - polymerase chain reaction; Tg: Transgenic; TUNEL: Terminal deoxynucleotidyl transferase dUTP nick end labeling; Wnt: Wnt signaling pathway.

## Competing interests

The authors declare that they have no competing interests.

## Authors’ contributions

JL performed experiments related to the Mdka gain- and loss-of-function experiments, collected the quantitative data, and performed the statistical analyses. RAU performed Id2a g-o-f experiments, collected the quantitative data, and performed the statistical analyses. SH was a rotating graduate student and performed *mdka in situ* hybridizations. A-AC generated antibodies used in the western blot analysis. JMG conceived of aspects of the study, participated in its design and coordination, and helped draft the manuscript. PFH conceived of the study, participated in its design and coordination, and helped draft the manuscript. All authors read and approved the final manuscript.

## Authors’ information

The senior authors, PFH and JMG, direct active laboratories that investigate the molecular mechanisms that govern eye and retinal development. Both are members of the Society for Neuroscience and the Association for Research in Vision and Ophthalmology. JL received a PhD in Physiology from the University of Wisconsin and is working as a postdoctoral fellow. A-AC was a graduate trainee at the University of Michigan and provided the original descriptions of *mdka* expression in the developing and adult retina of zebrafish. RAU and SH are graduate students at the University of Texas at Austin and University of Michigan, respectively. JMG and RAU recently published a paper in *Development* demonstrating the function of Id2a in the embryonic retina of the zebrafish.
